# IN-HOME MONITORING AND PRIVACY CONCERNS AMONG OLDER ADULTS

**DOI:** 10.1093/geroni/igz038.1358

**Published:** 2019-11-08

**Authors:** Moon Choi

**Affiliations:** KAIST Graduate School of Science and Technology Policy, Daejeon, Korea, Republic of

## Abstract

This study aimed to contribute to understanding older adults’ perspectives on privacy related to in-home monitoring by analyzing factors influencing the willingness to share their information collected by in-home monitoring systems. Study participants were interviewed at local senior centers in South Korea (N=170; mean age=77; 45.9% women). One out of eight (13.5%) participants did not want to share their information with anyone. Participants were more inclined to share their information with family and hospitals than with researchers, government agencies or insurance companies. The logistic regression results showed that participants living with others were more likely to share their information of vital signs, medication, and toileting compared to those living alone (ORs=20.5, 5.3, 4.5, respectively). Younger age and advanced ability to use the Internet were negatively associated with the willingness to share, overall. Information granularity and socioeconomic characteristics are important factors to consider when developing in-home monitoring systems for older adults.

